# High-resolution X-ray diffraction with no sample preparation

**DOI:** 10.1107/S2053273317008592

**Published:** 2017-06-29

**Authors:** G. M. Hansford, S. M. R. Turner, P. Degryse, A. J. Shortland

**Affiliations:** a Space Research Centre, Department of Physics and Astronomy, University of Leicester, Leicester LE1 7RH, England; b Celestijnenlaan 200E, Division of Geology, Centre for Archaeological Science, K.U. Leuven, Heverlee 3001, Belgium; c Centre for Archaeological and Forensic Analysis, DASSR/CDS, Cranfield University, Shrivenham, Swindon SN6 8LA, England

**Keywords:** energy-dispersive XRD, back-reflection geometry, sample preparation, non-destructive analysis, cultural heritage artefacts, synchrotron experiments

## Abstract

It is shown that energy-dispersive X-ray diffraction (EDXRD) implemented in a back-reflection geometry is extremely insensitive to sample morphology and positioning even in a high-resolution configuration. This technique allows high-quality X-ray diffraction analysis of samples that have not been prepared and is therefore completely non-destructive. The experimental technique was implemented on beamline B18 at the Diamond Light Source synchrotron in Oxfordshire, UK. The majority of the experiments in this study were performed with pre-characterized geological materials in order to elucidate the characteristics of this novel technique and to develop the analysis methods. Results are presented that demonstrate phase identification, the derivation of precise unit-cell parameters and extraction of microstructural information on unprepared rock samples and other sample types. A particular highlight was the identification of a specific polytype of a muscovite in an unprepared mica schist sample, avoiding the time-consuming and difficult preparation steps normally required to make this type of identification. The technique was also demonstrated in application to a small number of fossil and archaeological samples. Back-reflection EDXRD implemented in a high-resolution configuration shows great potential in the crystallographic analysis of cultural heritage artefacts for the purposes of scientific research such as provenancing, as well as contributing to the formulation of conservation strategies. Possibilities for moving the technique from the synchrotron into museums are discussed. The avoidance of the need to extract samples from high-value and rare objects is a highly significant advantage, applicable also in other potential research areas such as palaeontology, and the study of meteorites and planetary materials brought to Earth by sample-return missions.

## Abstract 2

A novel, high-resolution X-ray diffraction (XRD) technique that provides completely non-destructive, high-quality XRD analyses of unprepared samples is demonstrated. The method shows great potential in the characterization of cultural heritage artefacts.

## 1. Introduction   

When implemented in a back-reflection geometry with 2θ close to 180°, energy-dispersive X-ray diffraction (EDXRD) is uniquely insensitive to sample morphology and even to the precise positioning of the sample (Hansford, 2011 ▸). These characteristics open up the possibility of completely non-destructive X-ray diffraction (XRD) analysis of objects that have undergone no sample preparation at all. The back-reflection EDXRD technique inherently requires low-energy X-rays, up to approximately 6 keV, that have low penetrating power. It is therefore essentially a reflection-mode, surface-analysis XRD method, with typical penetration depths of a few microns. The 2011 paper considered the technique from a theoretical standpoint and with the aid of ray-trace modelling whereas subsequent work proved the claims experimentally (Hansford, 2013 ▸) and demonstrated a method to suppress fluorescence peaks in order to uncover overlapped diffraction peaks (Hansford *et al.*, 2014 ▸). All published work on this technique to date has focused on essentially low-resolution methods using solid-state X-ray detectors [silicon drift detectors (SDDs) and charge-coupled devices (CCDs)] to provide the energy dispersion. Implementation in this way enables a compact and lightweight instrument design suitable for handheld XRD instrumentation (Hansford, 2015 ▸). Nevertheless, it was recognized at the outset (Hansford, 2011 ▸) that the low resolution of diffraction peaks was a technological issue, not one that is fundamental to the technique itself. This paper describes the realization of the back-reflection EDXRD technique in a high-resolution configuration at the Diamond Light Source synchrotron in Oxfordshire, UK, and the results of the beamtime are presented.

The ubiquitous Bragg–Brentano geometry imposes strong constraints on sample positioning and the flatness of the sample surface because of the parafocusing nature of the geometry. Errors in either of these geometric parameters lead to instrument aberrations that adversely affect peak profiles and positions (see, for example, Wilson, 1963 ▸; Cheary *et al.*, 2004 ▸). However, there are alternative XRD geometries that offer relaxed constraints on the sample form and positioning. Some transmission XRD experiments are designed so that the XRD signal originates from within a well defined volume, known as tomographic energy-dispersive diffraction imaging (TEDDI) (Cernik *et al.*, 2008 ▸, 2011 ▸; Scarlett *et al.*, 2009 ▸; Lazzari *et al.*, 2009 ▸) and related techniques (Harding, 2009 ▸). This type of configuration can be used to perform three-dimensional mapping of the phase composition of samples or to probe specific regions in order to monitor processes *in operando*. Intense beams of high-energy X-rays are required for applications of this type which are therefore generally restricted to synchrotrons. In any case, there is an upper limit to the size of the specimen that can by analysed with these methods because of the need to transmit X-rays through the sample.

For reflection-mode geometries, parallel-beam XRD offers a significant degree of insensitivity to sample morphology and positioning (He, 2009 ▸). In this method the sample is illuminated with an approximately parallel beam of X-rays, prepared using a suitable optic such as a polycapillary lens or multilayer mirror, and the X-rays diffracted or scattered through a specific angle are selected with additional optics in the diffracted beam, such as crossed Soller slits (see, for example, Cheary *et al.*, 2004 ▸; Yamanoi & Nakazawa, 2000 ▸; Cao *et al.*, 2002 ▸; Wohlschlögel *et al.*, 2008 ▸; Misture & Haller, 2000 ▸). The use of parallel-beam optics in both the incident and diffracted beams ensures that only X-rays scattered through a defined 2θ angle are detected, irrespective of the point of interaction on the sample (as long as that point is within the field of view of the detection optics). Many modern laboratory diffractometers can be configured for parallel-beam XRD. As the method is an angle-dispersive XRD (ADXRD) approach it can suffer from sample shadowing problems, especially at low diffraction angles. If data are acquired in a θ–θ scanning mode the illumination of the sample changes during the scan, and this effect is greater for a sample with more pronounced morphology. In contrast, the geometry of the back-reflection EDXRD method essentially guarantees there can be no shadowing issues and the key parts of the experiment are static during data acquisition.

One advantage of parallel-beam XRD over the Bragg–Brentano geometry is that fewer geometric aberrations affect the instrumental line profiles which are generally Gaussian and independent of the scattering angle (Cheary *et al.*, 2004 ▸; Cao *et al.*, 2002 ▸; Welzel & Leoni, 2002 ▸). This characteristic simplifies line profile analysis and fitting, and the method is therefore particularly suited to microstructural analysis (Welzel & Mittemeijer, 2005 ▸). Parallel-beam XRD is commonly used for residual stress measurements because of the need to analyse manufactured parts, potentially with rough surfaces or complex geometries (Watkins *et al.*, 2003 ▸). The instrumental line shape of the back-reflection EDXRD technique is expected to be independent of energy, giving rise to similar advantages in microstructural and residual stress applications.

The most obvious application of back-reflection EDXRD in a high-resolution configuration is the analysis of cultural heritage objects. Examples of artefacts amenable to XRD analysis include archaeological pieces such as pottery (including pigments and glazes), jewellery, any objects made from stone or rock, and artworks such as paintings and sculptures. Studies of this sort are generally done for one of two reasons: either to answer questions related to provenance, giving insight into the material history of the objects, or to understand the stability and deterioration of materials in order to ensure proper conservation and to develop new conservation methods. Other potential application areas of back-reflection EDXRD are palaeontology and meteorite studies. A particularly interesting potential space-related application is the non-destructive analysis of materials provided by planetary sample-return missions, such as Martian, Lunar and asteroidal samples. In general, the method is suited to the analysis of objects that have high monetary or rarity value and that cannot be replicated or replaced. It is possible that there are industrial applications that conform to these criteria.

The primary aims of this study were to prove that the back-reflection EDXRD technique remains insensitive to sample morphology in a properly designed high-resolution configuration, and to gain insight into the characteristics of the method to inform further technique development including methods to analyse the resulting data. Experimental details are given in §2 of this paper, including a description of the beamline and the configuration specific to this study. The majority of the samples tested during the allocated beamtime were geological in nature, including a small number of fossil specimens. The methods used to process the data sets are described in §3, including the extraction and isolation of the diffraction signal in the presence of both X-ray fluorescence and Rayleigh scattering. The use of standards for *d*-spacing calibration is described in detail. Results are presented in §4, starting with demonstration of the insensitivity of the technique to the sample position. Various aspects of the analysis of the geological samples are highlighted. These include fitting of unit-cell parameters to gain insight into the materials, the advantages of the technique for analysis of unprepared phyllo­silicate samples and microstructural analysis. The results for a few, simple fossil samples are presented in §4.6. Although not the focus of this study, analysis of a small number of archaeological artefacts was attempted and the results are shown in §4.7. The experimental results and their implications for future work are discussed in §5, and the conclusions of this study and ideas for future work are presented in §6. The EDXRD spectra, diffraction line positions and assignments, and unit-cell parameter fits for all samples mentioned in this paper are available as supporting information.

## 2. Experimental details   

### 2.1. Beamline description   

All data were gathered on beamline B18 at the Diamond Light Source synchrotron. The electron beam at Diamond has an operating voltage of 3 GeV and a typical current of 300 mA. B18 is tailored for general-purpose X-ray absorption spectroscopy in the energy range 2.05–35 keV (Dent *et al.*, 2013 ▸), but could be readily adapted for energy-dispersive XRD. The X-rays at B18 are generated from a bending-magnet source. The beam is vertically collimated by a Si mirror coated with two metallic stripes, Pt for high energies and Cr for low energies, before passing through a double-crystal Si monochromator equipped with pairs of Si(111) and Si(311) crystals. A double-toroidal Si mirror located 25 m from the source serves to focus the beam horizontally and vertically, followed by removable harmonic rejection mirrors.

### 2.2. Experimental configuration   

**Figure 1:**
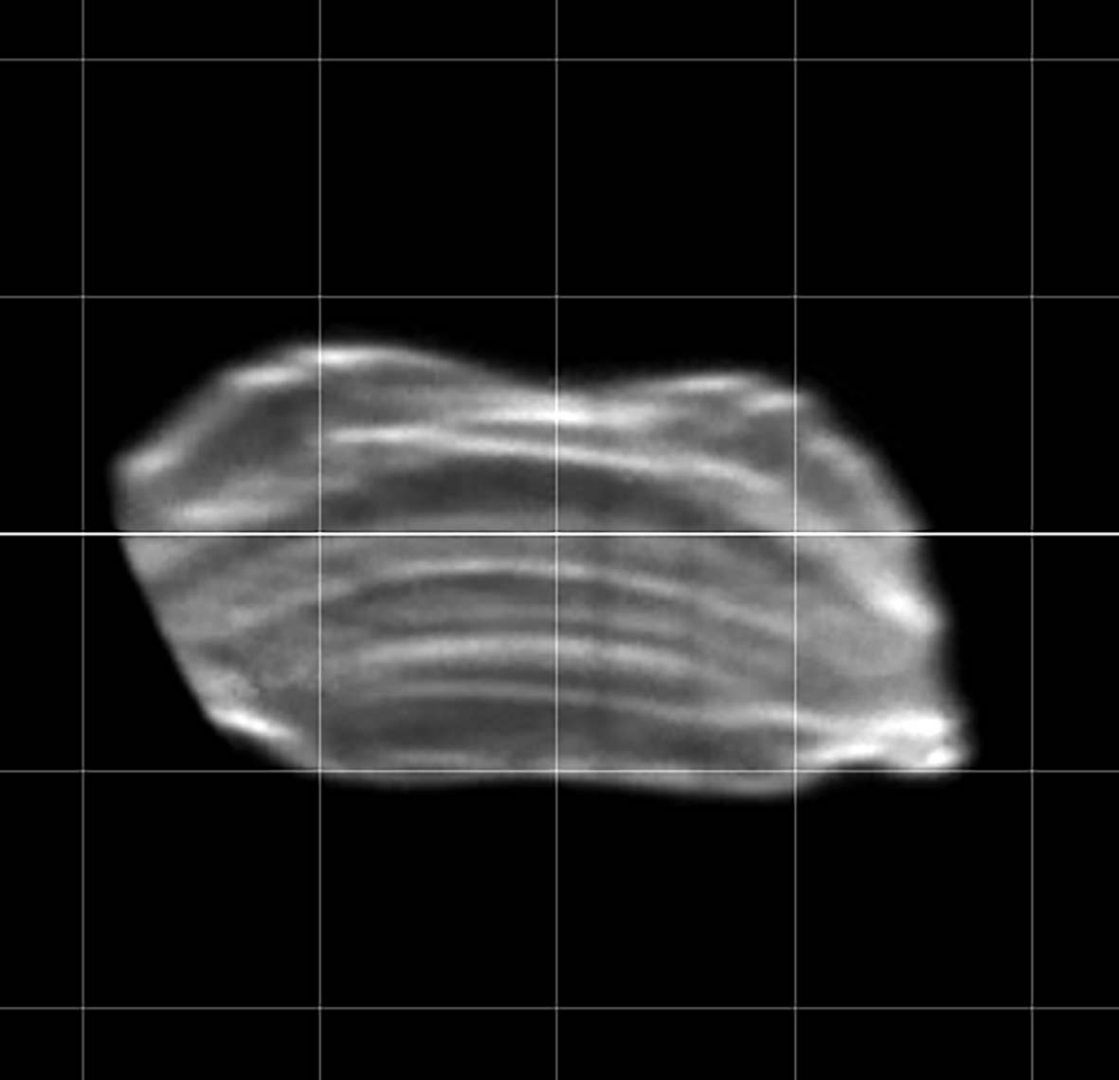
Image of a phosphor screen placed at the sample position and illuminated by the incident X-ray beam. The grid interval is 0.5 mm, calibrated by movement of the experimental table.

**Figure 2:**
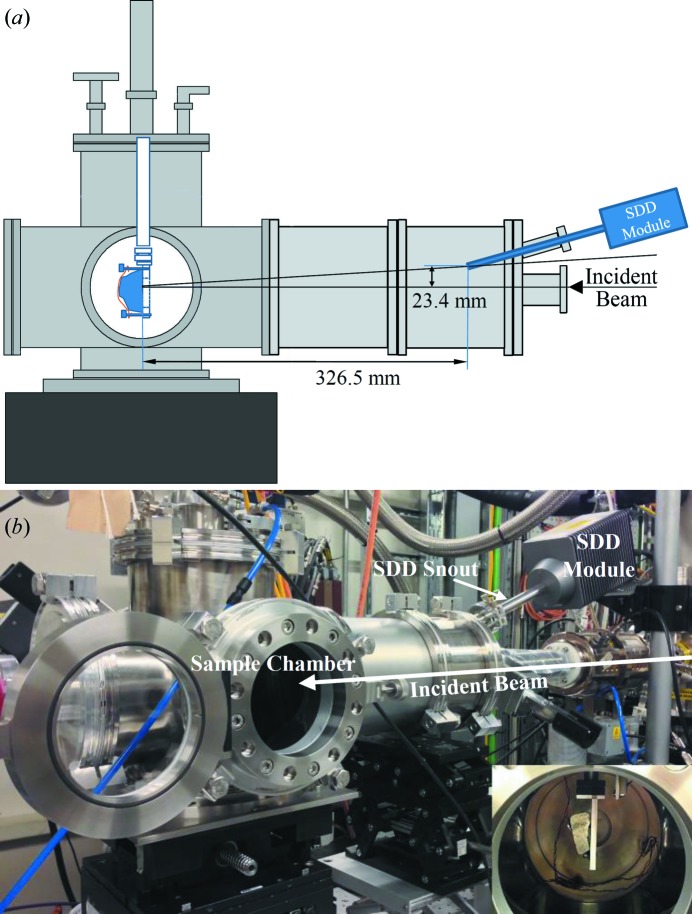
(*a*) Schematic diagram of the experimental configuration. The dimensions shown on the diagram are estimated values derived by a combination of measurement and extracting figures from engineering drawings and may have errors of a few mm. The normal to the SDD surface is inclined relative to the sample–detector vector by ∼28°, giving rise to a reduction in the SDD effective area of 12%. (*b*) Annotated photograph of the experimental configuration, roughly corresponding to the diagram in part (*a*). Inset: a photograph looking into the sample chamber, showing a rock sample attached to the kinematic mount.

The Si(111) double-crystal monochromator was used for the experiments reported here, giving an energy-resolution Δ*E*/*E* of 1.4 × 10^−4^, together with Ni-coated Si harmonic rejection mirrors. The pitch and roll of the double-toroidal mirror were adjusted to defocus the beam and give a suitable shape for the beam spot at the sample position, observed using a phosphor screen. The beam was trimmed slightly with horizontal slits to avoid hotspots. Fig. 1 ▸ shows an image of the phosphor screen with calibrated spatial scale; the spot size is approximately 1.7 × 0.9 mm (horizontal by vertical). The beam also passed through an ionization chamber prior to reaching the sample. Diffracted, fluoresced and scattered X-rays were captured by a 50 mm^2^ active-area Vortex-EM SDD mounted adjacent to the incident beam. The layout of the experiment is shown in Fig. 2 ▸ alongside a photograph. The calculated value of 2θ based on the dimensions given in Fig. 2 ▸(*a*) is 175.9°, but a more accurate value is derived in §3.3 using a *d*-spacing calibration standard.

Samples were rear-mounted onto a sample holder either with a simple clamp or, for smaller samples, with polyimide tape. The sample holder was secured in position in the main chamber with a magnetic kinematic mount, providing reproducibility in sample position. The chamber was sealed and then flushed by evacuating and re-filling with He several times in order to avoid fluorescence of Ar in air and to reduce attenuation of the low-energy X-rays used in these experiments. A residual amount of Ar is observable for some scans. He was used in the sample space rather than a vacuum because the chamber is shared with the windowless ionization chamber. The sample could be tilted about the vertical axis and multiple scans were performed for some samples over a range of tilt angles in order to observe the effects of, for example, preferred orientation of crystallites.

For each sample the monochromator was scanned continuously through the energy range 2.1 to 5 keV at 16.2 milli-degrees per data point, giving rise to an energy step size of 0.21 eV at 2.1 keV rising to 3.27 eV at 5 keV. The Vortex SDD and ionization chamber were hardware-triggered to acquire data simultaneously. The X-ray spectrum acquired by the SDD at each nominal monochromator energy was recorded. Thus, a large matrix of acquired counts was generated for each sample, with monochromator energy on one axis and SDD-detected energy on the other axis. Each scan was completed in 1376 s (approximately 23 min).

### 2.3. Samples   

The primary aim of accessing beamtime on B18 at Diamond was to develop the back-reflection EDXRD technique in a high-resolution configuration. Consequently, the majority of samples tested were pre-characterized geological samples, including rock specimens and pressed-powder pellets. These ranged from simple mono-mineral samples to more complex assemblages such as a basalt and samples containing clay minerals. Some samples were available in the form of an unprepared rock specimen and as a pressed-powder pellet derived from a portion of the same rock. A few fossil samples were tested as well as a limited number of archaeological samples.

For absolute calibration of *d* spacing, the NIST (National Institute of Standards and Technology) Si powder line position and line shape standard 640c (Freiman & Trahey, 2000 ▸) was used in the form of a pressed pellet. Pellets of quartz (SiO_2_) and corundum (Al_2_O_3_) powders were also useful as ‘secondary’ standards; see §3.3 for a full description of the use of these standards.

## 3. Data processing   

### 3.1. Extraction of EDXRD spectra   

A ‘quick-look’ spectrum was displayed during each monochromator scan, consisting simply of the summed counts of the SDD spectrum at each beam energy plotted live against energy. X-ray diffraction was observable as peaks, usually sharp, as the beam energy swept across diffraction lines. These peaks were situated on top of a rising baseline due primarily to sample X-ray fluorescence that grew in intensity as the beam energy increased. Jumps in the baseline were observed at elemental absorption edges, for elements present in the sample, because of the sudden appearance of new fluorescence peaks in the SDD spectra. The quick-look spectra were useful for a visual confirmation that the data acquisition was working as expected and for initial assessment of the results but were not used in subsequent data processing.

**Figure 3:**
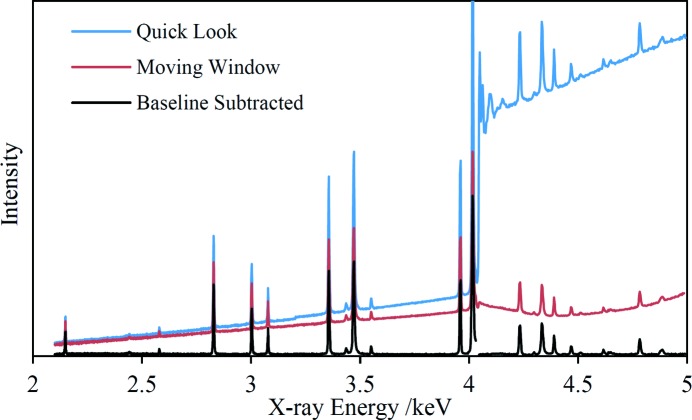
An illustration of the data processing steps for the extraction of the final EDXRD spectrum for a dolomitic rock sample. Full details are given in the main text.

The EDXRD spectrum of each sample was extracted from the data matrices in several steps, illustrated for a dolomitic rock sample in Fig. 3 ▸ which also shows the quick-look spectrum. It is interesting to note that this spectrum exhibits X-ray absorption fine structure (XAFS) above the Ca *K* absorption edge at 4038 eV due to variation of the Ca *K* fluorescence intensity; XAFS data were not used in subsequent analysis. In the first processing step the SDD spectrum at each beam energy was normalized using the ionization chamber signal, compensating for variations in the beam intensity at the sample. Apart from this normalizing step, diffraction peak intensities have been treated entirely qualitatively throughout the analysis and the intensity axis of each spectrum is essentially in arbitrary units. In the next step a moving window, centred at the beam energy, was used to extract the small part of each SDD spectrum containing the diffraction signal. This region of interest was summed to give a single data point in the EDXRD spectrum. The use of windowing serves to exclude most of the fluorescence signal in each SDD spectrum, but includes the diffraction and Rayleigh scattering signals as both processes are elastic. Different window widths were tested to find an optimum value. It was found that quite a small window width of 30 eV captured the greater part of the diffraction signal while simultaneously eliminating the interfering XAFS signal even quite close to absorption edges. Increasing the window width had only a very small effect on the signal-to-noise ratio of the diffraction peaks. Fig. 3 ▸ shows the output spectrum after windowing, illustrating these points. The baseline of this intermediate-stage spectrum is due primarily to Rayleigh scattering. There is a contribution from the Ca *K*β fluorescence peak above the absorption edge because this peak is not fully resolved from the diffraction/scattering peak in the SDD spectra until the scan reaches higher energies. The baseline is initially decreasing above the absorption edge because of a decreasing contribution from the Ca *K*β peak. In contrast, the Ca *K*α peak is entirely excluded by the moving window as it lies significantly below the absorption edge.

The varying baseline was removed in the final step. The spectrum was divided into sections according to the positions of any absorption edges present. For some samples such as the dolomite rock sample (Fig. 3 ▸), an additional break was introduced near 4.4 keV, avoiding diffraction peaks, because of the baseline curvature. Each section was fitted with a quartic polynomial in an automated iterative process in which data points lying above the fitted curve were excluded in the next iteration until convergence was achieved. An allowance for noise levels was made in order to exclude only diffraction peaks in each successive fit. Small 14 eV sections of the spectrum at each absorption edge were excluded because of the residual effects of XAFS on the spectrum. Lastly, a small bias level was added to avoid negative values in the final spectrum.

### 3.2. Peak fitting   

A software program has been written to fit a selection of line shapes to the peaks in the spectra in order to extract the centre line energy of each peak as accurately as possible. The available line shapes are: Gaussian, Lorentzian, pseudo-Voigt, Pearson VII and split-Pearson VII (Brown & Edmonds, 1980 ▸). For well resolved lines with good signal-to-noise, the Pearson VII line shape was found to reproduce the experimental peaks most accurately, though pseudo-Voigt profiles were almost as good in many cases. The pseudo-Voigt and Pearson VII profiles each require an additional fitted parameter per peak relative to Gaussian and Lorentzian profiles. For peaks with low signal-to-noise and/or that are overlapped, the fits using these profiles were sometimes unstable or produced un­physical parameter values. In these cases Gaussian or Lorentzian profiles were fitted. The split-Pearson VII profile was used for a small number of high signal-to-noise peaks with clear asymmetry.

### 3.3. Energy to *d*-spacing calibration   

Absolute calibration of the conversion from X-ray energy to *d* spacings is provided by the NIST Si powder (Freiman & Trahey, 2000 ▸). There are four diffraction peaks within the scanned energy range. As both the energies and the *d* spacings of these diffraction lines are known, they can be used to calibrate the experimental geometry using the Bragg equation cast in the energy domain:
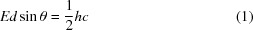
where *E* is the X-ray energy of the diffraction line, *d* is the corresponding *d* spacing, 2θ is the total scattering angle, *h* is Planck’s constant and *c* is the speed of light in a vacuum. The results of this geometry calibration are shown in Table 1 ▸. The average value for 2θ is 175.09° ± 0.14° which is in reasonable agreement with the geometry estimated by measurement and is taken to be the correct value in subsequent calculations. However, there is clear evidence of a downward trend in the derived 2θ values with increasing energy. This trend suggests that there is a discrepancy between the nominal beam energy and the true energy. A simple model was implemented to account for the discrepancy:
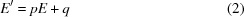
where *E* is now the nominal beam energy, *E*′ is the true beam energy, and *p* and *q* are parameters to be fitted. *E*′ can be substituted using the Bragg equation, giving
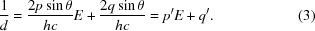
Fitting this equation to the data yields a direct conversion from the nominal beam energy to *d* spacing. Note that fitting the parameters *p*′ and *q*′ does not allow a refined estimate of the value of 2θ because the sin θ terms in equation (3) cannot be separated from *p* and *q*.

The four Si diffraction peaks could be used to derive values for *p*′ and *q*′ but because the lowest Si peak is at ∼3231 eV, the conversion of the lower energies in each scan to *d* spacings involves a significant extrapolation of the calibration that is unlikely to maintain the intrinsic experimental accuracy. To overcome this problem, the secondary quartz and corundum standards were used to constrain the calibration. These ‘standards’ do not have certified *d* spacings, but the relative positions of the diffraction peaks are strongly constrained by the fixed (but unknown) unit-cell dimensions, especially as both these minerals have high-symmetry trigonal crystal structures and their unit-cell dimensions can each be specified with just two parameters. The *d* spacings of quartz and corundum are given by
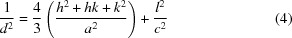
where *h*, *k* and *l* are the Miller indices of each diffraction peak and *a* and *c* are the unit-cell dimensions. A global fit of the Si, quartz and corundum diffraction peaks was performed based on equation (3); for the Si diffraction lines the *d* spacings reported in Table 1 ▸ were used, whereas for diffraction lines of the secondary standards equation (4) was substituted for the left-hand side of equation (3). Thus, six parameters were fitted simultaneously: *p*′, *q*′, *a*_Qz_, *c*_Qz_, *a*_Cor_ and *c*_Cor_, where the Qz and Cor subscripts represent quartz and corundum values, respectively. In addition to the four Si diffraction lines, 17 quartz and 11 corundum lines were included in the fit. A downhill simplex method (Press *et al.*, 2007 ▸) was used to fit the model to the data, based on minimization of the root-mean-square (r.m.s.) value of *d*_calc_ − *d*_fit_ where *d*_calc_ are the *d* spacings on the left-hand side of equation (3) (*i.e.* fixed values for Si; values calculated using the fitted unit-cell dimensions for quartz and corundum) and *d*_fit_ are the *d* spacings calculated on the right-hand side of equation (3). The results of the fit are shown in Table 2 ▸. The simplex fitting routine does not return error values, and the error of each parameter has been estimated as the change that gives rise to a 10% increase in the r.m.s. of the fit. The average value of |*d*_calc_ − *d*_fit_| for all 32 lines is 3.7 × 10^−5^ Å. The values of *p* and *q* have been calculated using the fitted parameters and assuming that 2θ = 175.09°; *p* is very close to unity and the offset *q* is a fraction of an eV, indicating that the nominal beam energy is very close to the true value as would be expected. Table 2 ▸ also reports average unit-cell dimensions of quartz and corundum derived from the 2015 release of the International Centre for Diffraction Data’s (ICDD’s) Powder Diffraction File (PDF) database (ICDD, 2015 ▸) (star-quality analyses at ambient temperature and pressure, with several outliers excluded in each case). The fitted unit-cell parameters in this work are in excellent agreement with the ICDD database values.

Several alternative models to the one specified by equation (3) were also tested including, for example, a quadratic in *E* and a model that assumed a linear error in the nominal monochromator crystal angle. However, none of the alternative models gave a significant improvement over the simple linear model represented by equation (3). It is also worth noting that the offset parameter *q*′ is required in order to achieve the stated accuracy; excluding this parameter results in a significantly poorer global fit, with an average |*d*_calc_ − *d*_fit_| value of 5.7 × 10^−5^ Å.

### 3.4. Analysis of sample data   

Each EDXRD spectrum was analysed by fitting line profiles to the diffraction peaks to extract accurate energies and converting these to *d* spacings using the calibration reported in §3.3. The mineralogical composition of some samples was known in advance *via* laboratory XRD characterization using a Bruker D8 Advance diffractometer. In these cases, assignment of the Miller indices of each line was essentially straightforward. In other cases, mineral identification and line indexing were attempted by performing *d*-spacing searches using the ICDD’s database and *SIeve+* program (Faber *et al.*, 2005 ▸). Using the assignments and associated *d* spacings, the unit-cell parameters of the corresponding mineral were fitted to the data. The purpose of these fits was firstly to confirm the identity of each mineral and that correct line assignments had been made, and secondly to glean additional information about the mineral such as its position within a solid solution series. Average values of |*d*_expt_ − *d*_fit_|, where *d*_expt_ are the experimentally derived *d* spacings, were typically in the range (1–4) × 10^−4^ Å. The higher values relative to the standards are consistent with generally broader peaks, lower signal-to-noise ratios and the inclusion of weak and partially overlapped lines in the analyses. Unresolved overlapped peaks were not included in the fits.

No attempt has been made to utilize peak intensities in the analyses. Intensities could in principle be used for phase quantification and structural analysis (such as determination of unit-cell atomic positions and occupancy factors) but only for those samples with good powder averaging. This point is discussed further in §5. A limited attempt to use peak widths to gain some microstructural insight has been made, see §4.5.

## 4. Results   

### 4.1. Insensitivity of back-reflection EDXRD to sample position   

**Figure 4:**
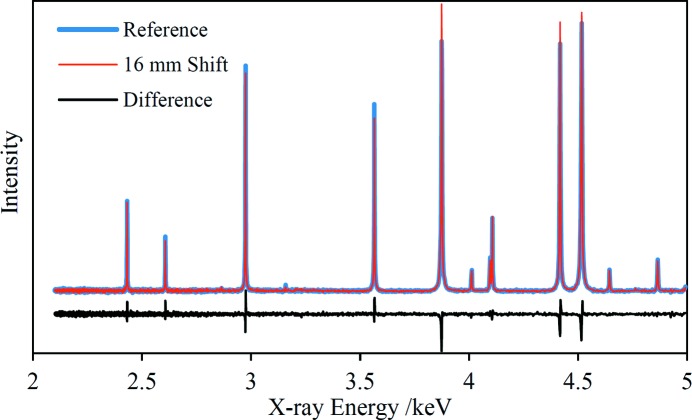
The EDXRD spectra of the corundum secondary standard recorded with the sample in the normal sample position and shifted away from the source and detector by 16 mm. The difference between the two spectra is shown in black. The difference spectrum has been vertically offset for clarity.

**Figure 5:**
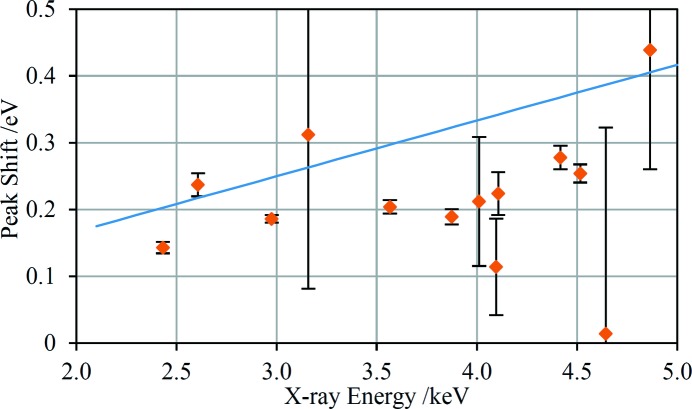
The measured shifts towards lower energies in the positions of the corundum diffraction peaks due to movement of the sample 16 mm away from the source and detector. The error bars are derived from the errors reported by the peak-fitting routine. The calculated shift is shown as a solid blue line.

The primary reason to implement the technique described in this paper is because it allows XRD analyses of samples independent of morphology and, therefore, without sample preparation in many cases. An important step in establishing insensitivity to sample morphology is proving insensitivity to the distance between the sample and the source and detector. With this aim in mind, the EDXRD spectrum of the corundum standard was acquired with the sample mounted in two different positions: the nominal position and with the sample shifted away from the source and detector by 16 mm. The two spectra are displayed in Fig. 4 ▸ along with the difference between them. The latter reveals slight shifts in the peak positions that are not otherwise discernible. To assess these shifts quantitatively, the peaks were fitted with Pearson VII profiles to extract positions. The differences in the peak positions are plotted in Fig. 5 ▸ against the nominal beam energy. The expected peak shifts can be calculated using the change in experimental geometry (Fig. 2 ▸) and its effect on 2θ. The total scattering angle increases by 0.228° which translates to peak shifts of Δ*E*/*E* = 8.33 × 10^−5^. Both the measured and predicted peak shifts are below 0.5 eV across the whole measured energy range and the trend of increasing shifts towards higher energies is approximately the same. Most of the measured peak shifts, particularly those with smaller associated error bars, lie below the prediction; the reason for this small discrepancy is not known. If a 16 mm sample shift was unaccounted for in the analysis, the error in the derived *d* spacings would also be Δ*d*/*d* = 8.33 × 10^−5^ which gives a maximum Δ*d* of 2.5 × 10^−4^ Å at a beam energy of 2.1 keV, decreasing to 1.0 × 10^−4^ Å at 5 keV. However, none of the samples analysed had surface morphology variation greater than ∼2 mm over the incident beam spot and so *d*-spacing errors arising from this effect are expected to be below the *d*-spacing accuracy of 3.7 × 10^−5^ Å determined in the calibration, §3.3.

### 4.2. Peak profiles   

A more detailed investigation of peak profiles was performed using the standards data. The 331 diffraction peak of the Si primary standard at ∼4980 eV was excluded because the high-side tail was curtailed at the end of the scan and because few points were recorded across the most intense part of the peak. The peaks of all three standards were most accurately reproduced with Pearson VII profiles, though pseudo-Voigt profiles were as good or nearly so in many cases. Some of the more intense peaks showed minor asymmetry with a longer tail on the low-energy side, particularly the corundum data sets which have higher signal-to-noise ratios. The intrinsic line shape of the experimental configuration may be slightly asymmetric with this effect observable only for the strongest peaks, or the asymmetry may be a sample-specific effect. The Pearson VII shape parameter, denoted *m*, derived from the peak fits showed significant differences between the standards. The Si primary standard peak fits had *m* ≃ 1.3 whereas the corundum peaks were best fitted with *m* ≃ 0.92, indicating a modest super-Lorentzian character; neither showed a significant trend with energy. In contrast, the quartz peak fits showed a trend of increasing *m* with energy, from ∼0.9 at 2.1 keV to ∼1.8 at 5 keV. The differences in the behaviour of the shape parameters of the three standards presumably reflect subtle microstructural differences in the materials. The peak widths of the standards have been used to estimate the instrumental contribution to peak broadening (see §4.5) and consequently it is not feasible to extract microstructural parameters for the standards.

### 4.3. Analysis of common, simpler minerals   

**Figure 6:**
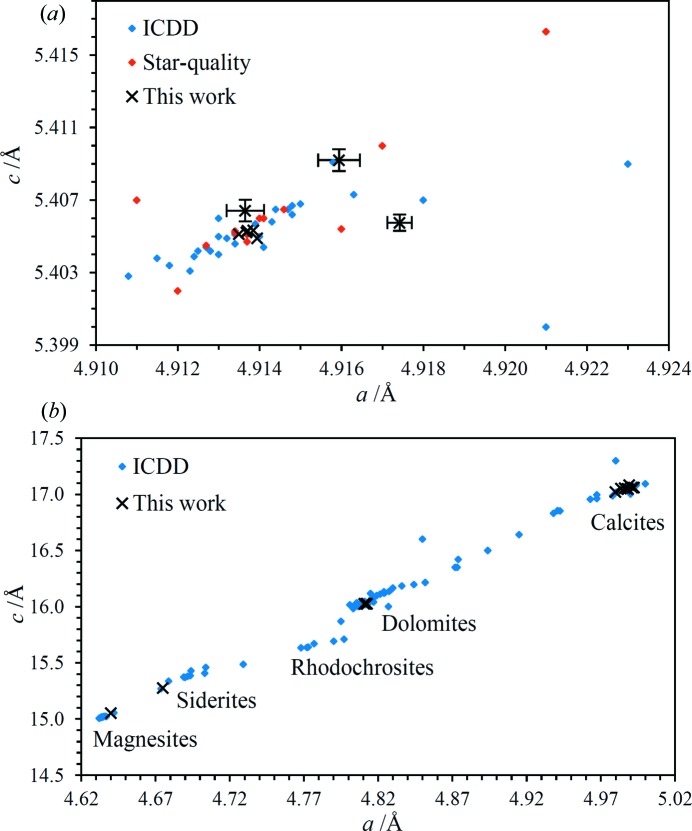
(*a*) A comparison of quartz unit-cell parameter determinations for this work (for samples where at least nine quartz peaks were identified – eight separate determinations) and derived from the ICDD database. Star-quality (the highest quality mark) ICDD data points are shown in red. The error bars are as reported by the fitting routine, but for five of the data points are not shown because they are smaller than the size of the symbols. (*b*) A comparison of unit-cell parameter determinations for a range of Ca-, Mg-, Fe-, Mn-containing carbonates (all belonging to the trigonal crystal system) for this work and derived from the ICDD database. The unit cells have been specified using hexagonal coordinates. This figure shows unit-cell dimensions for eight calcites, three dolomites [CaMg(CO_3_)_2_], one siderite and one magnesite as determined in this study. Rhodochrosite has the formula MnCO_3_.

Many of the samples analysed contain or are comprised of common minerals with relatively simple diffraction patterns. Assignment of Miller indices to the diffraction peaks was straightforward in these cases, leading to precise determinations of the unit-cell parameters. To exemplify these results, the unit-cell parameters of the quartz found in several samples are shown in Fig. 6 ▸(*a*) along with the corresponding parameters extracted from the 2015 release of the ICDD database (ICDD, 2015 ▸). Six of the eight quartz unit-cell determinations lie within or very close to the most dense clustering of points derived from the ICDD database. The right-most point corresponds to a chert sample consisting of cryptocrystalline quartz (see §4.5). The determination of the unit-cell parameters for this sample is presumed to be less precise than for most of the samples because the broader diffraction peaks give rise to greater uncertainty in peak positions and hence *d* spacings. The other two determinations with larger error bars are for samples with relatively minor quartz and most of the diffraction peaks have low signal-to-noise ratios. The differences in the unit-cell parameters of the two right-most points relative to the main cluster of points are nevertheless significantly greater than the estimated errors. It is noted in passing that the very close clustering of five of the quartz determinations in this work serves as evidence for the achievable accuracy reported in §§3.3 and 3.4.

Whereas quartz generally does not take part in solid solution series, carbonate minerals readily do so, leading to predictable correlations in the unit-cell dimensions as illustrated by the data in Fig. 6 ▸(*b*). As an example, there is a data point (this work) that lies close to the siderite (FeCO_3_) cluster of points but between the magnesites (MgCO_3_) and siderites. The ICDD data point that lies very close corresponds to a magnesian siderite (PDF #01-082-9278, Fe_0.65_Mg_0.35_CO_3_). It is very likely that the mineral observed in this work is also a magnesian siderite, based on the unit-cell dimensions.

### 4.4. Analysis of phyllosilicates   

**Figure 7:**
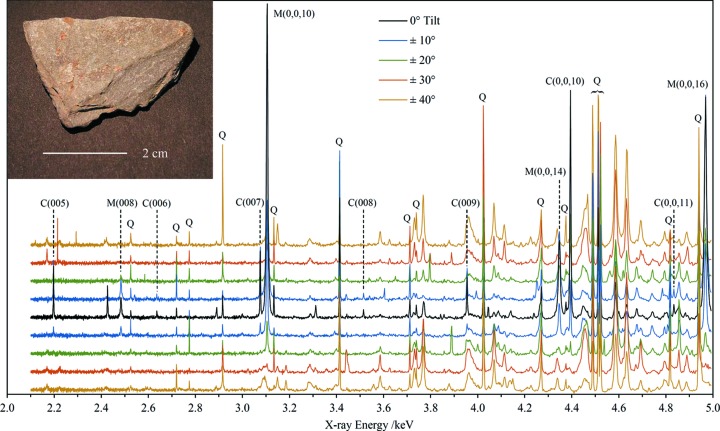
The EDXRD spectra of the mica schist specimen taken over a range of sample tilt angles (see main text for details). The spectra have been offset vertically for clarity. Quartz peaks have been labelled ‘Q’ and the identifiable mica (‘M’) and chlorite (‘C’) basal peaks have been labelled with their Miller indices. Other basal peaks are overlapped by other diffraction peaks or are too weak to be observed. Inset: photograph of the mica schist rock specimen.

XRD analysis of clay and phyllosilicate minerals, other than class identification *via* basal spacings, is notoriously difficult. Sample preparation, including crushing, grinding and separation of the clay fraction by a variety of methods, is time consuming and brings with it the danger of altering the minerals in some way (Moore & Reynolds, 1997 ▸). Typically, samples must also be prepared in multiple states such as oriented and random mounts, glycolation and dehydration by heating. Oriented mounts are the easiest to prepare but frequently show only basal diffraction peaks. Identification of specific polytypes can be difficult to achieve, and these issues are complicated by the occurrence of interstratified species and various types of disorder (Drits & Tchoubar, 1990 ▸). In the present experiments, the advantages of the back-reflection EDXRD method are illustrated for an unprepared clay-containing sample, visually identified as a mica schist (see Fig. 7 ▸), that exhibits a high degree of preferred orientation in its natural state. This sample contains mica, chlorite, quartz and minor amounts of other unidentified minerals, determined using the synchrotron data – no independent determination of the mineralogical composition of this sample has been made. The sample shows strong platy cleavage and was mounted with the cleavage plane perpendicular to the incident X-ray beam. In order to acquire diffraction data other than the basal peaks, additional spectra were acquired over a range of tilt angles at 10° intervals and up to 40° in each direction (see Fig. 7 ▸). The quartz in the sample is not expected to exhibit preferred orientation and indeed there is no correlation between the quartz peak intensities and the sample tilt angles. The quartz peaks do show some intensity variations from scan to scan, illustrating incomplete powder averaging for this mineral. These peaks are also very sharp relative to most other peaks in the spectra. The basal peaks of the two phyllosilicate minerals were straightforward to identify based on the regularity of the corresponding *d*-spacing series and, especially, the strong dependence of intensity on tilt angle. For example, the mica 0,0,10 reflection at 3105 eV is the most intense peak in the zero-tilt spectrum yet is virtually absent in the spectra acquired at 40° tilt angles. Using the intensity variation of this mica peak as a function of the tilt angle, the March parameter in the March–Dollase preferred orientation scheme (Dollase, 1986 ▸) has been estimated as *r* = 0.35 ± 0.02, confirming the high degree of orientation. In addition, there are many peaks that show the opposite trend, *i.e.* greater intensity at the higher tilt angles. Examples are the weak peaks at 2169 and 2223 eV, and peaks at 3149, 3732, 3768 and 4069 eV. There are also several examples of broad diffraction ‘bands’ that show the same tilt-angle dependence; the most prominent are located at approximately 3090, 3290, 3965 and 4455 eV. These bands have asymmetric, complex shapes that strongly suggest they cannot be interpreted as broadened individual diffraction peaks.

**Figure 8:**
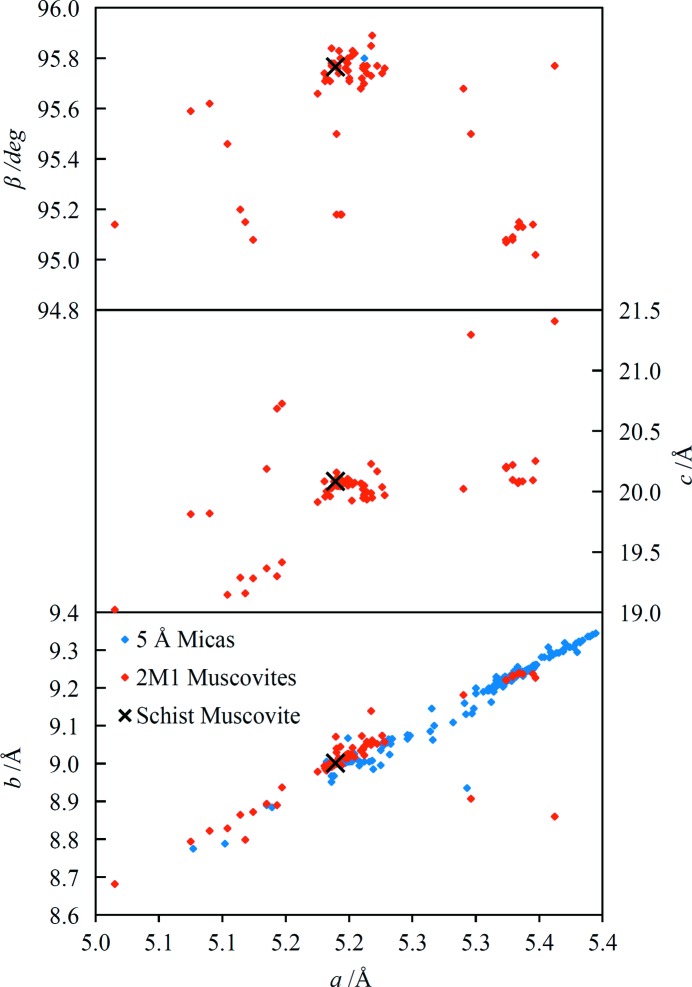
A comparison of the fitted unit-cell parameters for the schist muscovite with the corresponding values extracted from the ICDD database. The blue points represent all ICDD micas belonging to the monoclinic crystal system and with *a* close to 5 Å. The red points represent all ICDD structures specified as 2M1-muscovites.

The majority of the observed diffraction peaks, other than those due to quartz, are assignable to the mica. Assignment of the Miller indices of non-basal peaks was not straightforward and the additional information afforded by the dependence of intensities on tilt angle was crucial. Published tabulations of diagnostic diffraction lines for the identification of phyllo­silicate polytypes were also very useful (Bailey, 1980 ▸, 1988 ▸; Weiss & Wiewióra, 1986 ▸). Confidence in the correct assignment of the mica diffraction lines arises from the fit of the unit-cell parameters which incorporates a total of 35 lines with an average |*d*_obs_ − *d*_fit_| value of 1.6 × 10^−4^ Å, and the close agreement between the unit-cell parameters and ICDD database values. On the basis of the unit-cell parameters, the mica is a 2M1-muscovite. The unit-cell parameter fits for the three identified minerals are reported in Table 3 ▸ and the comparison of parameters with ICDD database values for the muscovite is shown graphically in Fig. 8 ▸. The unit-cell parameters lie within the main cluster of points representing 2M1-muscovites for all four parameters.

The positions of the basal peaks of the chlorite yield the combined unit-cell parameter *c* sin β = 14.1205 (4) Å. Using reasonable trial values for *a* and *b* it has not been possible to assign with any confidence the 20*l* lines commonly used for chlorite polytype identification (Bailey, 1980 ▸). However, the features described above as diffraction bands all lie close to positions predicted for ±1,3,*l* lines which are relatively intense in a randomly oriented mount (Bailey, 1988 ▸). The lack of clear 20*l* lines in the spectra suggests a significant degree of disorder in the chlorite structure (Bailey, 1988 ▸; Moore & Reynolds, 1997 ▸; Hayes, 1970 ▸) and it is believed that the appearance of the ±1,3,*l* features as diffraction ‘bands’ is directly related to this unspecified structural disorder. Further work is needed to confirm these conclusions and to suggest the type of disorder, particularly modelling of the effects on the EDXRD spectra. By assigning the ±1,3,*l* Miller indices to the maxima of the corresponding diffraction bands, and including the basal peaks and two weak lines identified as 060 and 262, a self-consistent unit-cell parameter fit results, reported in Table 3 ▸. Confidence in this fit is lower than for the mica, but nevertheless the derived unit-cell parameters are consistent with a 1MIIb-clinochlore.

### 4.5. Microstructural effects on peak widths   

**Figure 9:**
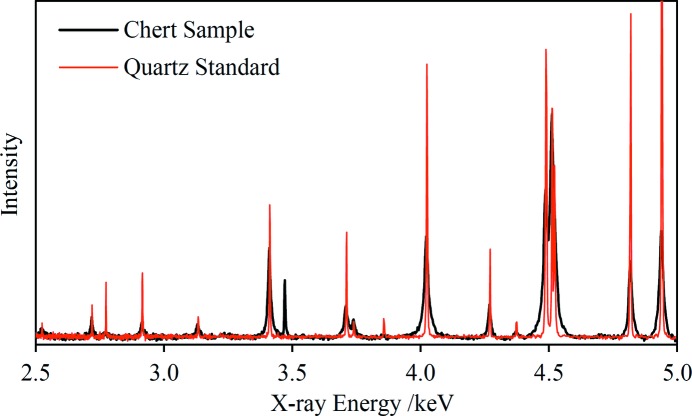
The EDXRD spectrum of an unprepared chert sample compared with the quartz secondary standard spectrum. The energy scale starts at 2.5 keV because no peaks were observed at lower energies. The vertical scale clips one of the quartz standard peaks in order to illustrate the remaining peaks with greater clarity.

Observed FWHM peak widths range from 1.6 eV up to ∼19 eV for the geological materials and including the standards. The standards have the narrowest peaks although there are some geological samples with comparable peak widths at the higher end of the energy scale. An approximate calculation of the expected widths based on the geometry of the experiment has been made, assuming negligible incident-beam divergence and including the effects of the finite beam spot size at the sample (Fig. 1 ▸) and the detector diameter. The width based purely on the geometry is assumed to add in quadrature with the monochromator passband [Δ*E*/*E* = 1.4 × 10^−4^ for Si(111)], though this factor increases the calculated widths by only 3%. The calculation is in good agreement with the experimental peak widths of the standards at lower energies with a minor divergence towards higher energies (the calculation giving lower values). It is reasonable to conclude that the peak widths of the standards are close to the limit allowed by the experimental set-up whereas the other specimens exhibit varying degrees of sample-dependent peak broadening, such as crystallite size and lattice strain effects. For example, Fig. 9 ▸ shows a comparison of the EDXRD spectra of the quartz standard and an unprepared chert rock specimen. The chert is expected to consist predominantly of cryptocrystalline silica (quartz), and indeed the diffraction peaks coincide with the quartz standard peaks but are significantly broader. This chert sample has the broadest peaks of any of the geological samples analysed in this work. There is also a much sharper peak at 3470 eV which is presumed to be a reflection from a crystallite of an unidentified mineral present within the chert.

In quantitative terms, the straight-line fit of the FWHM peak widths of the standards yields 1.03 eV at 2.1 keV beam energy, increasing to 4.55 eV at 5 keV. This instrumental resolution is equivalent to 0.015° at 2θ = 30.2° increasing to 0.083° at 2θ = 76.7° [see equation (5) of Hansford (2011 ▸)] for ADXRD using Cu *K*α radiation.

**Figure 10:**
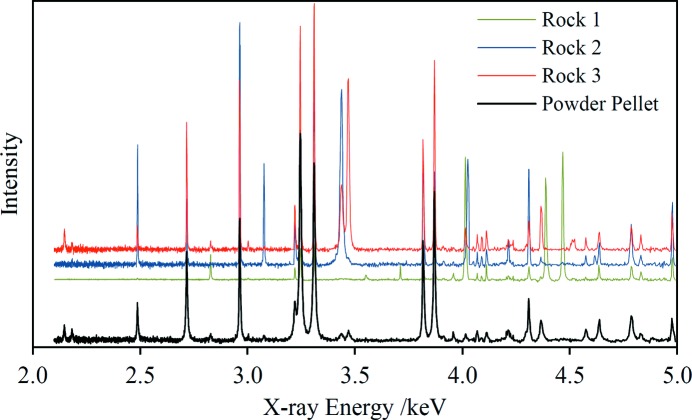
The EDXRD spectra of a limestone rock recorded at three different locations on the sample surface, and the spectrum of a pressed-powder pellet made from a portion of the same rock. The spectra have been offset vertically for clarity.

Peak broadening effects may be inadvertently introduced through sample preparation (Hill & Madsen, 2006 ▸). An example is presented in Fig. 10 ▸ which shows three EDXRD spectra of a limestone rock specimen, recorded at different locations on the same sample, and the spectrum of a pressed-powder pellet made from a portion of the same rock. This limestone contains calcite, dolomite and minor quartz [see Hansford *et al.* (2014 ▸), referred to as limestone A in that paper]. The diffraction peaks in the spectrum of the pellet are clearly broader, indicating the introduction of crystallite size and/or lattice strain effects during the pulverization and milling of the rock sample. Careful sample preparation is required to avoid these effects. The three rock spectra in Fig. 10 ▸ show significant variability in peak intensities, as well as the absence of some peaks in one spectrum that are present in another. These variations are believed to be caused both by inhomogeneity in the rock composition, suggested by visual inspection of the sample, and by incomplete powder averaging within the analysed volume.

The peak breadths of several samples with significantly broadened peaks, relative to the standards, have been assessed in the EDXRD equivalent of a Williamson–Hall (WH) plot (Williamson & Hall, 1953 ▸; Gerward *et al.*, 1976 ▸):
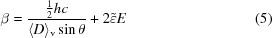
where β is the integral breadth due to the combined effects of crystallite size and lattice strain, 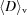 is the volume-weighted crystallite size and 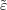 is some weighted average lattice strain (Delhez *et al.*, 1993 ▸). β values were calculated by subtracting the breadth due to the instrument alone from the experimental breadths. The instrument breadths were assumed to be equal to the values given by the standards; a straight line was fitted to the standards data to derive the energy dependence of the instrument breadth. The instrument and sample-dependent contributions to peak breadths are assumed to add directly rather than in quadrature because the peak profiles of both the standards and the samples are closer to Lorentzian than Gaussian (Scardi *et al.*, 2004 ▸; Delhez *et al.*, 1993 ▸). This issue is complicated by the fact that Pearson VII profiles describe the experimental peak shapes most accurately; the present analysis represents a simplification of more sophisticated analyses reported in the literature (for example, Langford, 1992 ▸; Mittemeijer & Welzel, 2008 ▸; Ungár *et al.*, 1999 ▸; Scardi *et al.*, 2004 ▸).

**Figure 11:**
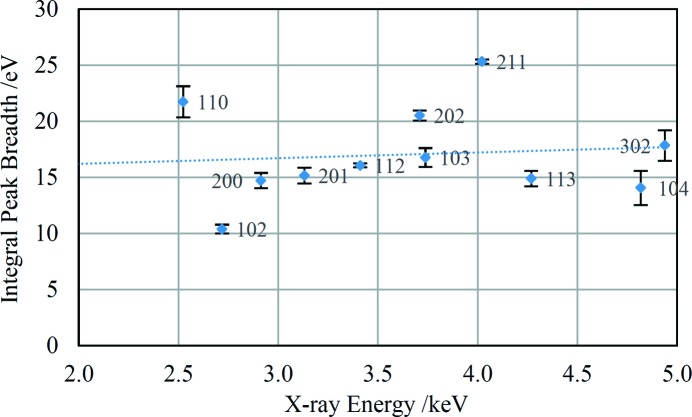
A Williamson–Hall-type plot for the unprepared chert rock sample. The Miller indices of each diffraction peak are shown on the plot and the error bars are as reported by the peak-fitting routine. The dotted line shows a straight-line fit through the points.

The WH-type plot for the chert sample is shown in Fig. 11 ▸. A straight-line fit through the points shows only a slight positive gradient, suggesting that microstrain is negligible for this sample. The intercept gives a volume-weighted crystallite size of 41 nm which is reasonable but should be regarded as semi-quantitative at best (Scardi *et al.*, 2004 ▸). The plot shows considerable anisotropy in the peak breadths, with no obvious dependence on the form of the Miller indices. An attempt was made to analyse the data assuming a cylindrical crystallite shape as described by Langford (1992 ▸) but the resulting plot did not support this interpretation. The exact nature of the anisotropy evident in Fig. 11 ▸ is not currently known.

**Figure 12:**
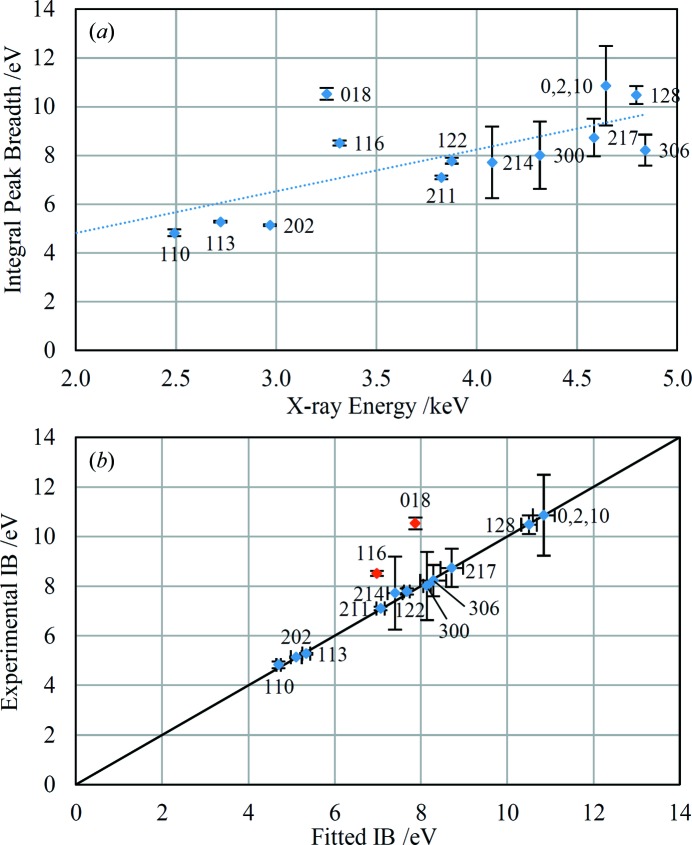
(*a*) A Williamson–Hall-type plot for the unprepared calcite rock sample. The Miller indices of each diffraction peak are shown on the plot and the error bars are as reported by the peak-fitting routine. The dotted line shows a straight-line fit through the points. (*b*) The experimental integral breadths (IB) for the same sample are plotted against the fitted values resulting from the Stephens anisotropic strain model (see main text for full details). Vertical error bars are as in part (*a*) whereas the horizontal error bars are as reported by the Stephens model-fitting routine. The points in red were excluded from the fit. The solid line shows the 1:1 correspondence (it is not a fit through the points).

Fig. 12 ▸(*a*) shows the WH-type plot for an unprepared rock sample retrieved from the Barrington Chalk Pit (Mortimore *et al.*, 2001 ▸). This sample is bright white in appearance, though does not have a chalky texture. The EDXRD spectrum shows the presence of calcite only. A straight line fitted through the points in Fig. 12 ▸(*a*) passes close to the origin, indicating that microstrain is the cause of the broadened peaks rather than crystallite size. Application of equation (5) yields a strain value of 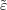 = 8.6 × 10^−4^. The scatter of the points about the best-fit line indicates anisotropy in the microstrain. The data for this sample have been reduced by application of a phenomenological model of anisotropic strain broadening based on crystal symmetry (Stephens, 1999 ▸). The energy-dispersive equivalent of Stephens’ equation (4) is
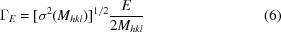
where Γ*_E_* is the integral breadth (after subtraction of the instrumental contribution) of each diffraction line, *M_hkl_* is defined as follows:
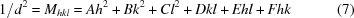
where *A*…*F* are metric parameters of the reciprocal lattice and σ^2^(*M_hkl_*) is the variance of *M_hkl_*. Constant factors relating to the use of integral breadth as a measure of peak width are absorbed into the *S_hkl_* parameters of Stephens. Stephens also introduced a parameter to interpolate between Gaussian and Lorentzian contributions to anisotropically broadened Voigt line shapes, but in this work the above equation has been applied without regard to the details of the observed line profiles which are best described with Pearson VII functions; application of equation (6) in this way represents a simplification of the Stephens model. Fig. 12 ▸(*b*) shows the experimental integral breadths plotted against fitted values derived by application of equation (6) to the calcite data. All the points except two lie on the 1:1 line within experimental uncertainties. The two outlier points correspond to the diffraction peaks 116 and 018 and, speculatively, the widths of these peaks may have an additional contribution from crystallite size effects if the crystallites are platy with the *c* axis perpendicular to the plates. Conversely, other diffraction peaks such as 0,2,10 would be expected to show a similar effect. The parameters fitted by the model, excluding 116 and 018, are: *S*_400_ = 4.11 (9) × 10^−5^, *S*_004_ = 2.8 (2) × 10^−7^, *S*_202_ = 6.6 (3) × 10^−6^ and *S*_301_ = −9.2 (6) × 10^−6^. Although these parameters are not directly related to physically meaningful microstructural parameters (Ungár *et al.*, 1999 ▸; Leineweber, 2011 ▸), the successful application of this model to the data lends support to the interpretation that anisotropic strain is the dominant peak broadening mechanism for this sample.

### 4.6. Fossil samples   

**Figure 13:**
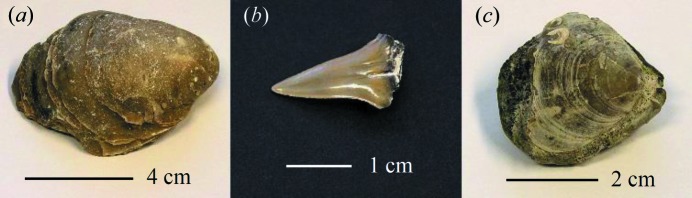
Photographs of the fossil samples: (*a*) oyster shell, (*b*) shark tooth, (*c*) brachiopod.

The non-destructive mineralogical analysis of fossil samples is a potential application of the back-reflection EDXRD method described in this paper, and is exemplified by the analysis of three common fossils. These fossils are: a Jurassic oyster shell from the Needingworth sand and gravel quarry, a shark tooth and a brachiopod, both Cretaceous and from the Barrington Chalk Pit (Mortimore *et al.*, 2001 ▸); images are shown in Fig. 13 ▸. All three fossils are quite simple mineralogically and the identification of the minerals present and indexing of the diffraction peaks were both straightforward. The results of unit-cell parameter fits are shown in Table 4 ▸.

**Figure 14:**
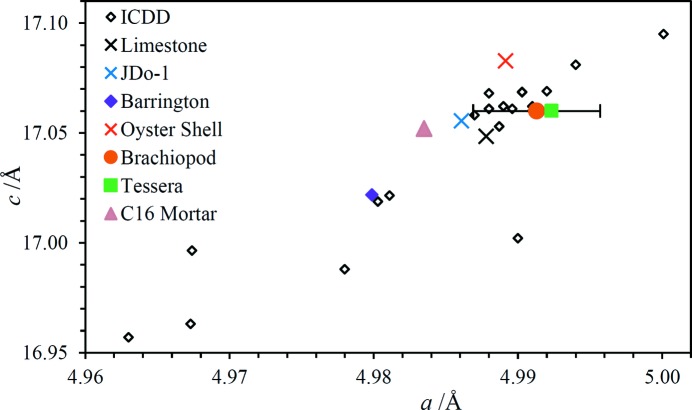
A comparison of calcite unit-cell parameter determinations for this work and derived from the ICDD database. All error bars are smaller than the sizes of the symbols except for the error in the *a* dimension of the brachiopod calcite. The latter has a much larger error because only four diffraction lines were observed in this case, and only two of those four have non-zero *h* and *k* Miller indices (lines 018 and 1,0,10). The unit cells have been specified using hexagonal coordinates. The sample names are as follows: limestone, refers to the pressed-powder pellet of the limestone rock sample mentioned in §4.5 (the EDXRD spectrum is shown in Fig. 10 ▸); JDo-1 is a pressed-powder pellet of the Japanese geological standard JDo-1 (Imai *et al.*, 1996 ▸; Hansford *et al.*, 2014 ▸); Barrington is the calcite rock sample retrieved from the Barrington Chalk Pit (see §4.5 and Fig. 12 ▸); oyster shell and brachiopod are the fossils described in §4.6 and shown in Fig. 13 ▸; tessera and C16 mortar are archaeology samples described in §4.7 and shown in Fig. 16 ▸.

For the oyster shell, all the observed peaks are assignable to calcite other than two weak peaks which are consistent with quartz. The peaks show large intensity variations, indicating poor powder averaging due to relatively large crystallites. The fitted unit-cell parameters are compared with ICDD calcite values in Fig. 14 ▸, along with calcites observed in other samples in this work. The point corresponding to the oyster shell lies a little above the main cluster of points though it is not known whether there is any particular significance to this observation.

**Figure 15:**
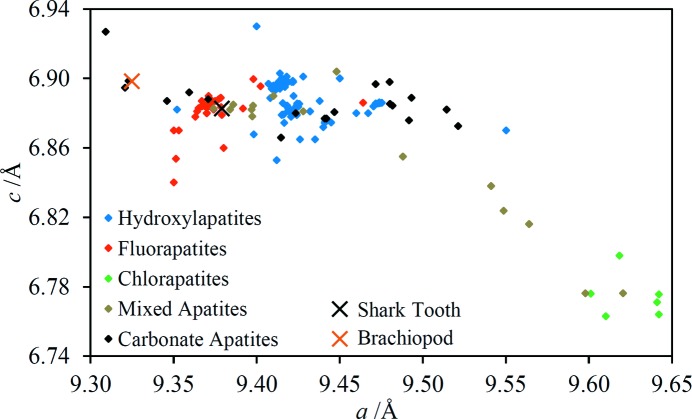
A comparison of apatite unit-cell parameter determinations for the shark tooth and brachiopod fossils (this work) and derived from the ICDD database. The points labelled hy­droxy­lapatite, fluorapatite and chlorapatite [Ca_5_(PO_4_)_3_Cl] are for minerals listed as pure whereas the mixed apatites have the general formula [Ca_5_(PO_4_)_3_(OH,F,Cl)], though they may have a composition close to one of the end-members of the solid solution. Carbonate apatites have the general formula [Ca_5_(PO_4_,CO_3_)_3_(OH,F,Cl)], but the carbonate content may be very low. Carbonate-containing apatites with relatively high carbonate content tend to have the lowest *a* unit-cell dimension for the structures in the ICDD database.

Almost all of the diffraction lines in the shark tooth EDXRD spectrum are assignable to fluorapatite [Ca_5_(PO_4_)_3_F], with just two lines with significant intensity remaining unidentified. The average value of |*d*_obs_ − *d*_fit_| for the fluorapatite unit-cell fit is somewhat worse than for the majority of analyses performed as part of this work, possibly because of the greater number of lines and the consequent potential for overlap. Nevertheless, the number of diffraction lines included in the fit lends confidence in the correctness of the line assignments. The unit-cell parameters are compared with ICDD database values in Fig. 15 ▸; the data point for the shark tooth lies much closer to the main cluster of fluorapatite points rather than the hydroxylapatites [Ca_5_(PO_4_)_3_OH] or any other apatites, consistent with expectations (for example, Kesmez *et al.*, 2004 ▸). The magnitude of the *a* unit-cell dimension suggests a fluorine content of 3.6 wt% based on the analysis of LeGeros & Suga (1980 ▸). The fluorine content of pure Ca_5_(PO_4_)_3_F is 3.77 wt%.

Two minerals have been identified in the brachiopod fossil: a carbonate-containing apatite and calcite. Although just four diffraction lines of calcite have been observed, three of these are the most intense peaks in the spectrum which suggests the calcite is present as relatively large crystallites. The unit-cell parameter fits are reported in Table 4 ▸ and shown graphically in Figs. 14 ▸ and 15 ▸. The comparison with ICDD-derived apatite unit-cell dimensions strongly suggests that the apatite mineral is carbonate-fluorapatite. The closest ICDD point in Fig. 15 ▸ corresponds to PDF #01-073-9696 which has a specified formula of Ca_4.95_(PO_4_)_4.96_(CO_3_)_1.283_­F_1.96_. This mineral is assumed to be a replacement mineral, in contrast to the oyster shell calcite and the shark tooth fluorapatite.

### 4.7. Archaeological samples   

High-quality, non-destructive phase analysis of archaeological samples is the primary anticipated application of the back-reflection EDXRD method. Spot analyses of several relevant samples were performed and the results are reported here. It is stressed that technique development was the primary focus of this study and the archaeological samples were chosen largely on an *ad hoc* basis.

#### 4.7.1. Sagalassos tesserae   

**Figure 16:**
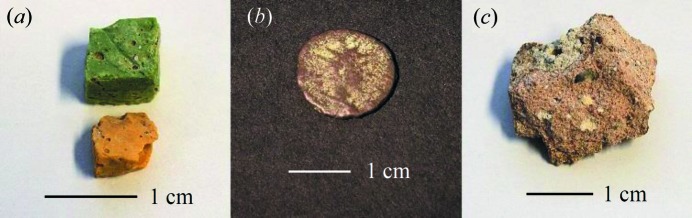
Photographs of the archaeology samples: (*a*) Sagalassos tesserae, (*b*) Roman coin, (*c*) 16th-century mortar.

Analyses were attempted for two sixth-century AD glass mosaic tesserae from the Roman baths complex at Sagalassos, south-west Turkey (Schibille *et al.*, 2012 ▸). Images of the tesserae are shown in Fig. 16 ▸(*a*). The EDXRD spectrum of the green tessera showed at best a couple of very weak diffraction peaks in an otherwise featureless spectrum. In some respects, this result is not surprising for a sample that consists predominantly of amorphous glass with relatively minor amounts of colourant materials. In contrast, however, the yellow tessera yielded a spectrum with a total of 18 clearly identifiable diffraction peaks. Assignment of these peaks to calcite and lead antimonate (Pb_2_Sb_2_O_7_, bindheimite) was straightforward. Calcium carbonate particles have previously been identified in Sagalassos tesserae, possibly derived from shell fragments in the sand used as the source of silica in the production of the tesserae (Schibille *et al.*, 2012 ▸). The lead antimonate imparts the yellow colour to the tessera. The unit-cell parameter fits are shown in Table 5 ▸; the calcite fit is shown graphically in Fig. 14 ▸. The unit-cell size of the lead antimonate, *a* = 10.4720 (5) Å, is significantly larger than the analyses listed in the ICDD database, such as PDF #00-42-1355 which has *a* = 10.4069 (4) Å. This discrepancy can be readily explained by the partial substitution of Sb by Sn and/or Fe (Schibille *et al.*, 2012 ▸; Lahlil *et al.*, 2008 ▸; Paynter & Kearns, 2011 ▸); for example, structures have been reported for Pb_2_Fe_0.5_Sb_1.5_O_6.5_ (PDF #01-077-2454) and Pb_2_SnSbO_6.5_ (PDF #04-013-3317) that have *a* = 10.4803 (2) Å and 10.5645 (2) Å, respectively. The relative intensities of the peaks assigned to lead antimonate are qualitatively consistent with calculated intensities, lending additional confidence in the identification of this mineral species.

**Figure 17:**
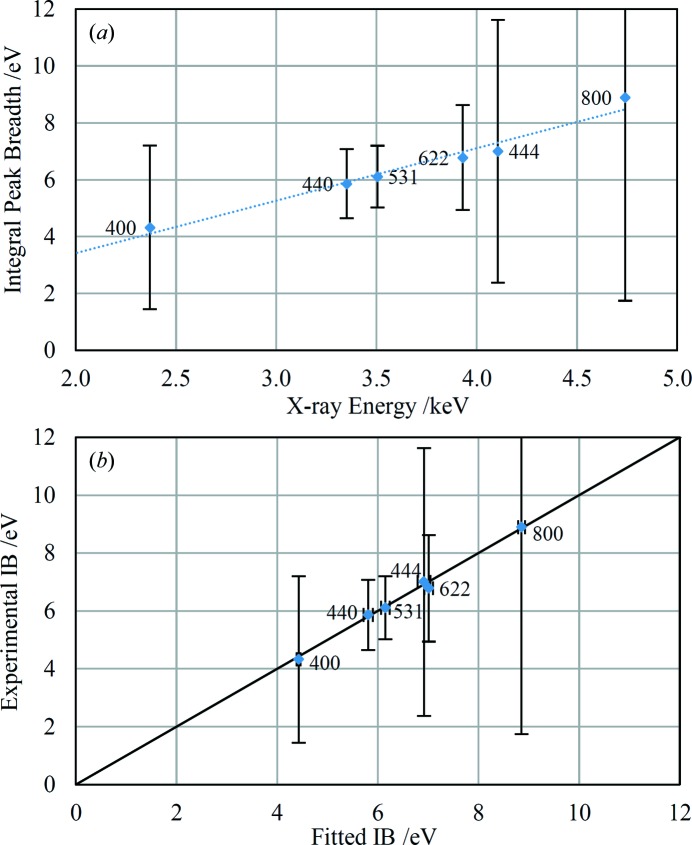
(*a*) A Williamson–Hall-type plot for the lead antimonate diffraction peaks of the yellow tessera. The Miller indices of each diffraction peak are shown on the plot and the error bars are as reported by the peak-fitting routine. The relatively large error bars are discussed in the main text. The dotted line shows a straight-line fit through the points. (*b*) The experimental integral breadths (IB) are plotted against the fitted values resulting from the Stephens anisotropic strain model. Vertical error bars are as in part (*a*) whereas the horizontal error bars are as reported by the Stephens model-fitting routine. The solid line shows the 1:1 correspondence (it is not a fit through the points).

Several of the lead antimonate diffraction peaks are sufficiently strong to allow a microstructural analysis, as described in §4.5. The WH-type plot is shown in Fig. 17 ▸(*a*); the relatively large error bars derived from the peak profile fits are due to consistent asymmetries in the peaks which have longer tails on the high-energy sides. Fitting split-Pearson VII peak profiles gave very similar peak widths but with comparable or larger uncertainties, presumably because of the greater number of parameters being fitted. Speculatively, the asymmetry may be caused by variation in the Fe and Sn content of the lead antimonate structure leading to a range of unit-cell sizes. The straight-line fit in Fig. 17 ▸(*a*) passes close to the origin, strongly suggesting that peak broadening is caused by microstrain rather than crystallite size. Application of equation (5) yields a strain value of 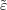 = 9.2 × 10^−4^. The anisotropy in the observed peak widths is quite small, but nevertheless the Stephens model has been applied to the data as in §4.5; the resulting fit is shown in Fig. 17 ▸(*b*) and the fitted parameters are *S*_400_ = 1.161 (18) × 10^−6^ and *S*_220_ = 1.67 (9) × 10^−6^. This fit reduces the average discrepancy between observed integral breadths (after subtraction of the instrumental contribution) and the fitted breadths from 0.21 eV for the straight-line fit in Fig. 17 ▸(*a*) to 0.09 eV for the Stephens model fit, suggesting that the latter genuinely explains the minor anisotropy in the peak widths.

#### 4.7.2. Roman coin   

Two spot analyses were attempted for the Roman coin shown in Fig. 16 ▸(*b*). This coin comes from a private collection; the location of origin is unknown and the date is estimated to be first to third centuries AD. One analysis spot was located at the centre of the head side of the coin and the second spot was located on an area showing a green patina on the same side. The corresponding EDXRD spectra are included in the supporting information and are subsequently referred to as the ‘centre’ and ‘green’ spectra. Cuprite (Cu_2_O) gives the most intense peaks in both of the spectra although elemental copper can also be identified. Quantitative X-ray fluorescence (XRF) data show that this coin contains 96.0% Cu, 1.5% Ag, 1.0% Pb, 0.44% Sn and other elements ≤ 0.2% (percentages quoted as wt%); the XRD and XRF data are clearly consistent with each other.

The unit-cell parameter fits are shown in Table 5 ▸. The unit-cell sizes for cuprite determined at each of the two analysis spots are very similar to each other and with values in the ICDD database [*e.g. a* = 4.2685 (5) Å for PDF #04-007-9767]. The data for copper are more problematic. Firstly, there are only three diffraction lines occurring in the recorded spectral range. The highest energy of the three lines lies very close to a cuprite line, and for the ‘centre’ spectrum is only a shoulder on the cuprite peak for which a reliable position could not be determined. In the ‘green’ spectrum the other two copper lines are rather weak. The copper peaks are also quite broad in both spectra which reduces the accuracy with which their positions can be determined. For these reasons only two lines contribute to each of the unit-cell fits. The derived unit-cell dimensions are a little different from each other and also larger than the value for pure copper (*a* = 3.615 Å, PDF #00-004-0836). It is presumed that some of the minor elements present are incorporated into the copper lattice, altering the lattice spacing [for example, Cu_0.99_Pb_0.01_ has *a* = 3.634 Å (Savitsky *et al.*, 1982 ▸)].

The cuprite peaks of the ‘centre’ spectrum have shoulders on the low-energy side and for the two lowest-energy peaks it was possible to fit peak positions, yielding a unit-cell dimension of *a* = 4.288 (2) Å assuming cubic symmetry and assignment of the same Miller indices as the adjacent cuprite peaks. This unit-cell size and symmetry are consistent with wüstite (FeO), but only 0.2 wt% Fe was measured by XRF and this explanation is considered unlikely. These additional peaks could be due to a second, distinct cuprite phase containing impurities that alter the lattice spacing.

There remain at least 15 additional diffraction peaks for which phase identification has not been possible. Some of these peaks are seen in both spectra. Five of the peaks are accurately consistent with a cubic phase with *a* = 11.1038 (4) Å. However, the only plausible mineral assignment is arsenolite (As_2_O_3_) but the XRF results show that only 0.2 wt% As is present. The ‘green’ spectrum was acquired in the hope of detecting phase(s) specific to the patina but it has not been possible to identify any candidates.

#### 4.7.3. 16th-Century lime mortar   

A small piece of 16th-century lime mortar, Fig. 16 ▸(*c*), was recuperated from between the bricks of the ancient city wall around Antwerp, in Flanders (Belgium), by Antwerp city archaeologists. The mortar was subjected to analysis by the back-reflection EDXRD method, and quartz, hematite (Fe_2_O_3_) and calcite were readily identified. However, many of the calcite peaks are clearly clusters of two or even three closely spaced peaks, direct evidence for the presence of several distinct calcites in the mortar. Consistent indexing of the peaks in order to extract the unit-cell parameters of the calcites proved to be problematic despite extensive efforts. A key difficulty was that peak intensities could not reliably be used to aid assignment of peaks to structures with different unit-cell parameters because of incomplete powder averaging. Ultimately, the peaks were assigned to four different calcites but it is stressed that the assignments are not secure and it is not claimed that there are definitely four distinct calcites present. The unit-cell parameter fits are reported in Table 5 ▸, but only one calcite, for which confidence in the line assignments is highest, is included. It has not been possible to make an assignment for approximately 11 further diffraction peaks across the spectral range. It is expected that further progress with the analysis of this sample could be made with the acquisition of data at lower energies (higher *d* spacings) and using methods to improve powder averaging (see §5).

## 5. Discussion   

Many unprepared geological samples have been analysed by back-reflection EDXRD as part of this study as well as a small number of fossil and archaeology samples. The calibrated *d* spacings extracted from the spectra were used to precisely fit unit-cell parameters in each case, as long as the phase(s) present could be identified and the peaks indexed. These analyses demonstrate that all the observed diffraction peaks of any given phase are found in the positions predicted by the small number of unit-cell parameters to a high degree of accuracy, despite the non-uniform sample morphology and the lack of sample preparation. Together with confirmation that movement of the sample away from the source and detector (§4.1) and tilting of the sample (§4.4) both have a negligible effect on the positions of diffraction peaks, these analyses constitute proof that the back-reflection EDXRD technique can successfully be applied in a high-resolution configuration to many samples completely non-destructively and without any preparation of the sample (Hansford, 2011 ▸).

A limitation of performing no sample preparation is that good powder averaging cannot be guaranteed. By ensuring that relative peak intensities are representative, good powder averaging is desirable both as an aid to phase identification and peak indexing, and to allow the application of whole-pattern-fitting methods for phase quantification (Scarlett *et al.*, 2009 ▸) and for other purposes such as structure refinement using Rietveld methods. The 16th-century lime mortar sample (§4.7.3) provides a good example where improved powder averaging would help greatly. Nevertheless, it is possible to perform phase identification even when some diffraction peaks are missing from the spectra in favourable cases. Phase identification is frequently all that is required for archaeological purposes, the primary intended application of the technique.

Some unprepared samples have crystallites that are sufficiently small to ensure good powder averaging in any case, but for those that do not there are several ways to mitigate this issue in future work. The beam footprint on the sample could be enlarged and the sample could be moved laterally during data acquisition in order to probe a greater volume of the sample and increase the number of crystallites exposed to the beam. Both these methods would reduce the spatial resolution in mapping applications. The sample could also be spun, as in conventional laboratory XRD, about the axis perpendicular to the sample surface. An annular detector (Hansford, 2011 ▸) could replace the spot detector used in these experiments which, in terms of improving powder averaging, is equivalent to spinning the sample (assuming that the incident X-ray beam is perpendicular to the sample surface). In the experiments described here, the circular detector intercepts an azimuthal angular range of only 19.5° at most of the Debye–Scherrer diffraction rings. Consequently, use of an annular detector would immediately increase the number of crystallites observed by a factor of 18 or more. An annular detector would also serve to maximize the XRD signal while conforming to the angular constraints of the method. Finally, the sample could also be dynamically tilted about one or more axes perpendicular to the incident beam during data acquisition. This method is not used in focusing geometries because it introduces aberrations and broadens the diffraction peaks.

For any given sample, the X-ray penetration depth is differential across the useful energy range, a factor that applies to reflection-mode EDXRD in all cases. If the sample is homogeneous up to the maximum penetration depth then there is little consequence. The probed volume of the sample is smaller at lower energies which could in principle adversely affect powder averaging towards lower energies. These considerations have been discussed further in Hansford (2011 ▸).

Significant improvements in the future implementation of the back-reflection technique in a high-resolution configuration can be made based on the results of the present study. A key change would be to extend data acquisition to lower energies in order to capture a greater range of *d* spacings. Larger *d* spacings are considerably more diagnostic for phase identification purposes because of the lower density of diffraction peaks. Together with improvement in powder averaging using the methods described above, it is expected that phase identification and peak indexing will both become significantly easier for the more challenging and complex samples. Working at lower energies presents a challenge because of the absorption of X-ray flux by windows in the beam path and by any path length in air. For smaller samples which can be subjected to a vacuum or a He atmosphere, mounting within a chamber offers the best opportunity to extend the energy range down to at least 1 keV. For larger samples which cannot feasibly be mounted inside a chamber, it would be possible to continuously flush a small gap between the X-ray beam aperture and the sample in order to reach 1 keV in energy. In back-reflection EDXRD, this energy is equivalent to 14.3°2θ for ADXRD using Cu *K*α radiation. The set-up employed in the present study could also have been improved by use of an annular detector mounted closer to the sample. This change would have boosted the XRD signal allowing the observation of weaker diffraction peaks and/or faster data acquisition, the latter being highly advantageous for mapping applications. Future work will also ensure that the spacing of points in the acquired spectra is more even (see §2.2).

There are many possible ways to implement the back-reflection EDXRD method in a high-resolution configuration. A key design question is what part of the experiment provides the energy dispersion. In the present experiments, the beamline Si(111) monochromator was primarily responsible for the achieved resolution of diffraction peaks. The SDD played a secondary role in excluding the majority of the XRF signal from the sample, except near absorption edges. It would also be possible to illuminate the sample with a broadband X-ray source and use, for example, a monochromator or analyser crystals in the diffracted beam in a wavelength-dispersive set-up. A particularly interesting option, and one that would enable a laboratory implementation rather than relying on synchrotron facilities, would be to employ superconducting transition-edge sensor (TES) arrays (Ullom *et al.*, 2014 ▸; Ullom & Bennett, 2015 ▸) for X-ray detection and to provide good energy resolution. These sensors admit an especially simple conceptual design for the experiment as a whole, and the simultaneous acquisition of the whole EDXRD spectrum would avoid the need for time-consuming energy scanning, although the operation of the sensors is significantly non-trivial (Fowler, 2016 ▸). Whatever design concept is chosen for future experiments, geometrical broadening of diffraction peaks must be taken into account alongside the resolution afforded by the energy-resolving element of the experiment. In the present study, the ultimate resolution was limited by the geometry rather than the monochromator; for example, the diffraction peak width due to geometry alone is calculated to be ∼1.7 eV at 3 keV compared with the monochromator resolution of 0.42 eV. If the detector was moved closer to the incident beam so that 2θ = 178°, for example, and with no other changes, the geometrical broadening would improve to 0.71 eV at 3 keV. The detector could also have been moved closer to the sample in order to increase the XRD signal, though there is a trade-off with geometrical broadening because the range of 2θ angles seen by the detector will increase. In all cases, mounting the detector as close as possible to 2θ = 180° minimizes geometrical broadening and maximizes insensitivity to sample morphology and position.

There is no fundamental reason why back-reflection EDXRD cannot be used to perform any of the structural analyses that can be carried out using conventional XRD methods. For example, Rietveld refinement has been demonstrated for synchrotron EDXRD, though a significant investment of effort is required to establish the method for any given experimental configuration (Scarlett *et al.*, 2009 ▸; Madsen, 2015 ▸). Microstructural analysis was demonstrated in this study in §§4.5 and 4.7.1. The analyses presented are relatively simplistic, mainly because the instrument response was insufficiently characterized. Future work in this area should focus on running several standards over a wider energy range, and on testing with samples with well characterized microstrain and crystallite size properties. Nevertheless, the microstructure results presented here show that the back-reflection technique has considerable promise in extracting microstructural information on samples in their natural state, avoiding any possibility of changes induced by sample preparation. Furthermore, the method may be especially suited to this type of analysis for the same reason that parallel-beam XRD is also advantageous, *viz*. the independence of the instrumental contribution to the line shape on energy (Welzel & Mittemeijer, 2005 ▸). Extraction of microstructural parameters has the potential to provide a fingerprint of the state of a material in archaeology (Ungár *et al.*, 2003 ▸).

Regarding the analysis of phyllosilicate minerals (see §4.4), it is a notable achievement that it has been possible to confidently assign the diffraction peaks of a muscovite, comprising part of a mica schist sample, despite the fact that no *d* spacings larger than 2.95 Å were recorded (corresponding to 30.3° 2θ for Cu *K*α radiation). Furthermore, this analysis was done for a natural sample not prepared in any way and containing several minerals. Normally, the observation of large *d* spacings at low angles in ADXRD is crucial in the analysis of clays and phyllosilicates. The technique presented here completely avoided the need for sample preparation, including separation of the clay fraction and the preparation of multiple sample states such as random and oriented mounts, glycolation and dehydration by heating. Clays by definition are very fine-grained, typically <2 µm, and good powder averaging is therefore essentially guaranteed. The high-resolution back-reflection EDXRD technique appears to be highly suited to the characterization of clay-containing samples. For samples with a high degree of crystallographic texture (whether natural or induced by sample preparation), the acquisition of data over a range of sample tilt angles is a powerful method to access and distinguish diffraction peaks with different diagnostic characteristics [*e.g.* 00*l* basal peaks, polytype diagnostic peaks (Bailey, 1980 ▸, 1988 ▸; Weiss & Wiewióra, 1986 ▸), *k* = 3*n* chlorite peaks (Moore & Reynolds, 1997 ▸)], analogous to the preparation of random and oriented mounts using conventional preparation methods. The separation of different types of diffraction peaks in this way reduces crowding and overlap. It would be very interesting in future work to extend the tilt angle beyond 40° (see Fig. 7 ▸) up to 90° and observe this type of sample ‘edge on’. This would allow effective access to diffraction peaks with small and zero *l* indices, considerably aiding peak indexing and subsequent analysis, *e.g.* observation of 060 peaks for the distinction of dioctahedral and trioctahedral varieties (Moore & Reynolds, 1997 ▸). The analysed mica schist sample also contains chlorite and there is good evidence of considerable structural disorder for this phase. Further work to characterize disorder in phyllosilicates and other lamellar structures (Drits & Tchoubar, 1990 ▸) is expected to be an interesting avenue for future research.

The non-destructive analysis capabilities of the back-reflection EDXRD technique are suited to the study of fossils as it is clearly advantageous to keep the specimens intact, especially for rare examples. A simple demonstration of mineral identification and the derivation of crystallographic parameters was presented in §4.6 for several common fossils, intended to illustrate the potential of the technique in this field of study. The ability of the method to answer palaeontologically relevant questions will depend on the details of each specimen and the purpose of the study. Mapping the mineralogical variation across a fossil sample will undoubtedly be important in some cases. As with all XRD mapping applications, there will be a tension between keeping the analysis spot size small to maximize spatial resolution and achieving sufficient powder averaging to enable phase identification and crystallographic analysis.

Analysis of a small number of archaeological samples, chosen essentially on an *ad hoc* basis, was also attempted, §4.7. The most significant success was the unambiguous identification of the colourant species lead antimonate in one of the Sagalassos tesserae. Furthermore, the unit-cell size was accurately derived and shown to be consistent with earlier studies which suggested partial substitution of Sb by Sn and/or Fe, and microstructural information was also extracted from the data. Several crystallographic phases were identified for the Roman coin and the 16th-century lime mortar. It is expected that future work with these samples over a wider *d*-spacing range and with greater signal-to-noise ratios would yield further insights into the composition of these artefacts, such as the identification of the green patina on the coin. The data for the mortar sample yield persuasive evidence for the presence of at least three distinct calcite phases, though it was not possible to make a definitive assignment of the individual diffraction peaks. This sample provides a good example of the limits of the technique, as implemented in this study, particularly in respect of samples with poor powder averaging due to large crystallites. It is likely that a more complete analysis would be possible by implementing the methods to improve powder averaging described above, combined with extension to lower energies/larger *d* spacings.

The results presented here for the archaeological samples, together with anticipated future technical improvements, illustrate the considerable potential of the back-reflection EDXRD technique in providing high-quality XRD data for cultural heritage studies completely non-destructively. The identification of crystallographic phases along with the extraction of microstructural parameters for samples in their natural state can be expected to help greatly in the provenancing of artefacts. For example, stone artefacts are notoriously difficult to provenance, particularly if sampling is not allowed. There are however large collections of reference materials derived from ancient quarries and stone sources available for comparison of XRD results. The back-reflection EDXRD technique could also be used to identify deterioration products found on a range of different cultural heritage objects, such as bronzes. Knowledge of the specific break-down products is a crucial factor in the formulation of effective conservation strategies.

As described above, the high-resolution back-reflection EDXRD technique can be implemented in the laboratory through the use of superconducting TES arrays, providing a possible mechanism to move the technique directly into museums. This approach would have great advantages in terms of avoiding the costly and risky movement of artefacts, many with high financial and/or rarity value, out of the museum. At the present time, TES arrays are very expensive and operationally complex (Fowler, 2016 ▸), but both these barriers to implementation can be expected to lessen as further research and development effort is invested in these devices (Ullom & Bennett, 2015 ▸).

## 6. Conclusions   

The study presented in this paper demonstrates that it is possible to obtain XRD data of very high quality on geological and archaeological samples with no preparation of the samples at all using the back-reflection EDXRD technique. The key criterion to ensure insensitivity to sample morphology and positioning is the use of a 2θ scattering angle as close to 180° as feasible. Phase and polytype identification, derivation of precise unit-cell dimensions and extraction of microstructural information were all illustrated as part of this study. Furthermore, there is every reason to suppose that other types of XRD-based analysis, such as residual stress measurement, Rietveld refinement and quantitative phase analysis, can be implemented using the back-reflection technique with the appropriate investment in adapting the data processing algorithms. Whole-pattern-fitting methods and quantitative analysis impose constraints on the degree of powder averaging, as for any powder diffraction technique, and consequently not all unprepared samples can be analysed using such methods. The back-reflection EDXRD technique is inherently a surface-analysis method and this factor may be a limitation for some samples. The primary application of the technique is likely to be in the field of cultural heritage studies for which the avoidance of the need to prepare samples in any way is an overwhelming advantage. Many such studies would benefit simply from the most basic of XRD capabilities, phase identification. On this basis, it is expected that a very wide range of heritage objects are amenable to meaningful analysis using high-resolution back-reflection EDXRD. More sophisticated analyses involving, for example, the extraction of compositional information by establishing the position of a phase within a solid solution series or the extraction of microstructural parameters would be appropriate for some subset of artefacts. The technique is applicable whenever a sample has high financial or scarcity value and should not be altered in any way; examples are given in §1.

Future work will focus on extending the energy range of the acquired EDXRD spectrum to lower energies in order to access reflections corresponding to larger *d* spacings which have considerable diagnostic value for phase identification and peak assignment purposes. There is a trade-off between the XRD signal and the spectral resolution of diffraction peaks that must be considered in any specific configuration. Positioning of the detector as close to 2θ = 180° as feasible is always an advantage in the back-reflection technique and the use of an annular detector would maximize the signal and improve powder averaging by acquisition of the XRD signal from a greater number of crystallites. Future microstructural studies would benefit greatly from more detailed characterization of the instrumental response through the use of several standards, including those with known microstructural characteristics. Further synchrotron-based studies are appropriate to continue the transition from technique development to more meaningful and systematic cultural heritage studies.

A longer-term aim is the establishment of a facility employing a superconducting TES array for both X-ray detection and energy dispersion. Excepting the complexity of the TES array itself, a set-up of this type is especially simple conceptually and imposes very relaxed constraints on the power of the X-ray source and on the geometrical tolerances on relative positioning of the component parts of the experiment. A particular advantage is that the whole spectrum would be acquired simultaneously. Some simple calculations and simulations strongly suggest that spectra with good signal-to-background can be acquired in just a few minutes, opening up mapping applications. Furthermore, simultaneous and co-located XRF data would also be acquired. The high resolution would ensure minimal overlap of fluorescence and diffraction peaks.

## Supplementary Material

A brief description of each sample mentioned in the article, the processed EDXRD spectra, d-spacings with line assignments and unit-cell parameter fits.. DOI: 10.1107/S2053273317008592/sc5103sup1.xlsx

## Figures and Tables

**Table 1:** Geometry calibration results using the NIST Si powder data

Line assignment	Energy† (eV)	*d* spacing (Å)	Derived 2θ
220	3231.193	1.920217	175.226°
311	3788.913	1.637567	175.224°
400	4569.993	1.357799	174.994°
331	4980.195	1.246002	174.910°
		Average =	175.09°

† Pearson VII fit.

**Table 2:** Energy to *d*-spacing calibration results

Parameter	Fitted or derived value†	ICDD star-quality average‡
*a*_Qz_	4.91394 (11) Å	4.9141 (13) Å
*c*_Qz_	5.40490 (22) Å	5.4055 (18) Å
*a*_Cor_	4.75921 (13) Å	4.7597 (9) Å
*c*_Cor_	12.9921 (6) Å	12.9937 (27) Å
*p*′	1.611436 (20) × 10^−4^ eV^−1^ Å^−1^	
*q*′	8.2 (6) × 10^−5^ Å^−1^	
*p*	0.999897 (12)	
*q*	0.51 (4) eV	

† Error estimates are given in parentheses and quoted in units of the least significant digit.

‡ Standard deviations in parentheses. See text for details of the selected analyses.

**Table 3:** Unit-cell parameter fits for the unprepared mica schist sample

Parameter†	Quartz	Mica	Chlorite
*a*	4.91350 (8) Å	5.1891 (5) Å	5.368 (3) Å
*b*		9.0020 (12) Å	9.3024 (11) Å
*c*	5.40512 (14) Å	20.0839 (7) Å	14.2239 (7) Å
β		95.766° (4)	96.934° (17)
No. of lines	15	35	13
Average |*d*_obs_ − *d*_fit_|	5 × 10^−5^ Å	1.6 × 10^−4^ Å	1.4 × 10^−4^ Å

† Error estimates are given in parentheses and quoted in units of the least significant digit.

**Table 4:** Unit-cell parameter fits for the minerals found in the fossil samples

Fossil sample:	Oyster shell	Shark tooth	Brachiopod	
Parameter†	Calcite	Fluorapatite	Calcite	Carbonate-fluorapatite
*a*	4.98915 (20) Å	9.3796 (7) Å	4.9913 (44) Å	9.3248 (4) Å
*c*	17.0828 (9) Å	6.8825 (8) Å	17.0600 (17) Å	6.8985 (6) Å
No. of lines	15	39	4	34
Average |*d*_obs_ − *d*_fit_|	1.4 × 10^−4^ Å	4.7 × 10^−4^ Å	2.2 × 10^−4^ Å	1.6 × 10^−4^ Å

† Error estimates are given in parentheses and quoted in units of the least significant digit.

**Table 5:** Unit-cell parameter fits for the minerals found in the archaeology samples Each entry consists of: unit-cell parameters, number of lines included in the fit, average |*d*_obs_ − *d*_fit_|. Error estimates are given in parentheses and quoted in units of the least significant digit.

Phase and crystal system	Yellow tessera	Roman coin	C16 mortar
Lead antimonate (cubic)	*a* = 10.4720 (5) Å		
	11, 2.2 × 10^−4^ Å		
Calcite (trigonal)	*a* = 4.9923 (4) Å		*a* = 4.98349 (21) Å
	*c* = 17.060 (3) Å		*c* = 17.0520 (20) Å
	6, 2.9 × 10^−4^ Å		8, 1.4 × 10^−4^ Å
Quartz (trigonal)			*a* = 4.9137 (5) Å
			*c* = 5.4064 (6) Å
			9, 2.5 × 10^−4^ Å
Hematite (trigonal)			*a* = 5.0304 (3) Å
			*c* = 13.7395 (16) Å
			5, 1.2 × 10^−4^ Å
Cuprite (cubic)		Centre:	
		*a* = 4.26850 (8) Å	
		4, 8 × 10^−5^ Å	
		Green:	
		*a* = 4.2692 (4) Å	
		4, 4.6 × 10^−4^ Å	
Cu (cubic)		Centre:	
		*a* = 3.6252 Å†	
		2, 5 × 10^−6^ Å	
		Green:	
		*a* = 3.6186 Å†	
		2, 3.9 × 10^−4^ Å	

† The error estimates returned by the fit are not considered reliable because only two lines are used to deduce one unit-cell dimension.
